# Access to Health Care in Contexts of Livelihood Insecurity: A Framework for Analysis and Action

**DOI:** 10.1371/journal.pmed.0040308

**Published:** 2007-10-23

**Authors:** Brigit Obrist, Nelly Iteba, Christian Lengeler, Ahmed Makemba, Christopher Mshana, Rose Nathan, Sandra Alba, Angel Dillip, Manuel W Hetzel, Iddy Mayumana, Alexander Schulze, Hassan Mshinda

## Abstract

The authors present a framework for analysis and action to explore and improve access to health care in resource-poor countries, especially in Africa.

Access to health care is a major health and development issue. Most governments declare that their citizens should enjoy universal and equitable access to good quality care. However, even within the developed world, this goal is difficult to achieve, and there are no internationally recognized standards on how to define and measure “equitable access” [1]. Evidently, big disparities exist between the poor and the better off with respect to access to health care services and health status [2–4]. Gaps in child mortality between rich and poor countries are wide, as well as between the wealthy and the poor within most countries. Poor children are not only more likely than their better off peers to be exposed to health risks and have less resistance to disease, they also have less access to preventive and curative interventions. Even public subsidies for health frequently benefit rich people more than poor people. Clearly, more of the same is not enough [3]: To improve equitable access, innovative and community-based approaches are needed to better align health care services with poor people's needs, expectations, and resources.

This article presents a framework for analysis and action to explore and improve access to health care in resource-poor countries, especially in Africa. The framework links social science and public health research with broader development approaches to poverty alleviation. It was developed in the frame of the ACCESS Programme, which focuses on understanding and improving access to prompt and effective malaria treatment and care in rural Tanzania as an empirical case study [5,6]. The article first provides a brief outline of three approaches to investigating health care access, focusing either on health seeking, health services, or livelihoods. It then presents a framework that combines the three approaches, exemplified with research findings and interventions of the ACCESS Programme.

## Access to Health Care from Three Perspectives

Health-seeking studies focus on people [7–10]. They apply pathway models and follow sick persons step by step from the recognition of symptoms through different types of help seeking until they feel healed or capable of living with their condition. Health-seeking studies provide a deeper understanding of why, when, and how individuals, social groups, and communities seek access to health care services, and investigate interactions between lay persons and professionals [11]. In this perspective, social actors are the potential driving force for improving access to effective and affordable health care, but they are often constrained by politics and the economy on national and international levels [12–14].

Health service studies concentrate on factors influencing access to health care, which they commonly define as utilization rates [15–17]. They apply determinants' models and consider access as a general concept summarizing a set of more specific dimensions, such as availability, affordability, accessibility, adequacy, and acceptability. Although they take into account demographic characteristics of health service users, their knowledge about the disease, and, more recently, wealth as measured by household assets, health services studies tend to pay more attention to the supply than the demand side [18,19]. They search for policy interventions to reduce supply barriers and improve the delivery of services, including availability of health facilities, equipment, and qualified staff, staff skills, protocols of diagnosis, treatment, and quality of care. Moreover, they are less oriented towards health-seeking processes. Interventions on the demand side are commonly limited to information, education, and communication (IEC) campaigns.

Livelihood approaches—as the name implies—emphasize assets (including material and social resources) and activities needed to gain and sustain a living under conditions of economic hardship [20–25]. Access is a key issue for sustainable livelihoods [26]. Recent studies applying the Sustainable Livelihood framework of the United Kingdom Department for International Development to study HIV/AIDS [27] and malaria (J. Chuma, unpublished PhD thesis) demonstrate the many difficulties people face in gaining access to household and community assets and how this constrains their strategies to cope with the disease. In other words, not only possession, but mobilization of household and community assets is a critical factor influencing people's access to health care and other health-related services. Interventions target communities and social groups, emphasize solidarity and empowerment, and try to improve livelihood conditions.

## Access to Health Care with a Livelihood Focus

**Figure 1:**
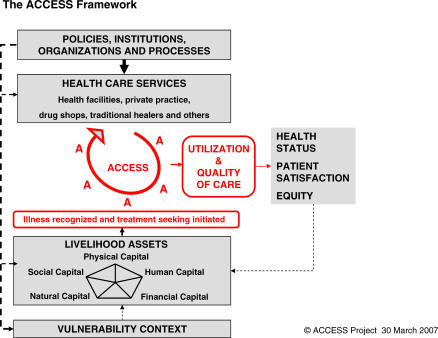
The Health Access Livelihood Framework Once people recognize an illness and decide to initiate treatment, access becomes a critical issue. Five dimensions of access influence the course of the health-seeking process: Availability, Accessibility, Affordability, Adequacy, and Acceptability. What degree of access is reached along the five dimensions depends on the interplay between (a) the health care services and the broader policies, institutions, organizations, and processes that govern the services, and (b) the livelihood assets people can mobilize in particular vulnerability contexts. However, improved access and health care utilization have to be combined with high quality of care to reach positive outcomes. The outcomes can be measured in terms of health status (as evaluated by patients or by experts), patient satisfaction, and equity.

The Health Access Livelihood Framework combines health service and health-seeking approaches and situates access to health care in the broader context of livelihood insecurity (Figure 1).

## Five Dimensions of Access

Access becomes an issue once illness is recognized and treatment seeking is initiated. Five dimensions of access influence the course of the health-seeking process: Availability, Accessibility, Affordability, Adequacy, and Acceptability (see Table 1).

**Table 1:**
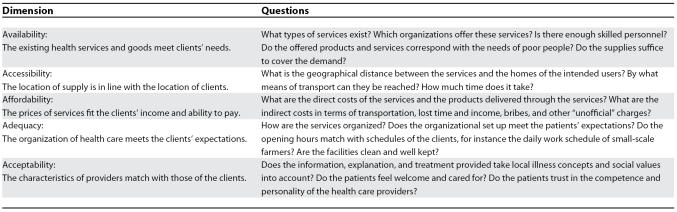
Five Dimensions of Access to Health Care Services

A review of literature from Tanzania found, for instance, that people considered the availability of essential drugs a prerequisite to the credibility of health services [28]. Problems of accessibility, including long distances to nearest dispensary or health center, scarce public transport, and lack of bicycles and other private means continued to be major access barriers. Issues related to affordability were also major obstacles: complaints about fees were frequent, and even if official fees were exempted (e.g., for children under five) or waived (e.g., for persons temporarily unable to pay), people often ended up paying for drugs, small charges, kerosene, and even ambulance transport. Poor people had to resort to short-term coping strategies like selling critical assets such as crops to pay for health care, especially in times of emergencies. Adequacy and acceptability in terms of people's judgment of quality of care also played an important role.

What degree of access is reached along the five dimensions depends on the interplay between (a) the health care services and the broader policies, institutions, organizations, and processes (PIOP) [29] that govern the services, and (b) the livelihood assets people can mobilize and combine in particular vulnerability contexts. Hence, access improves as health care services become better aligned with clients' needs and resources.

## The Health Care Services and the PIOP

Sick persons and caregivers seek help not only in health facilities or private practice, but also in drug shops and pharmacies as well as from healers representing a wide array of medical traditions. Access to these health care service providers is governed by cultural norms, policies, laws and regulations, which themselves are influenced by broader trends in society, global health policy, research, and development.

In malaria control, for instance, the World Health Organization has increasingly recognized the role of the private retail sector in improving access to prompt malaria treatment, since self-treatment at home is often the first response to a malaria episode [30]. The National Malaria Control Programme of Tanzania also acknowledges the importance of shops for home management of malaria [31]. A shop survey of the ACCESS Programme showed, however, that the proportion of general shops with antimalarials in stock had dropped from 27% in 2001 [32] to 8% in 2004 [5]. The reduced availability of antimalarials in general shops was largely due to a change in the policy of the Ministry of Health. Until 2001, chloroquine was the first-line antimalarial and was treated as an over-the-counter drug; Part II drug stores—a category of shops below pharmacies—were allowed to sell chloroquine and, in practice, chloroquine was also tolerated in general shops, where it was widely available [32]. After the policy change from chloroquine to sulphadoxinepyrimethamine (SP) as the first-line antimalarial in 2001, SP remained classified as prescription-only. The Tanzania Food and Drugs Authority (TFDA), which is responsible for all regulatory aspects of drugs and other medical products in the country, did not reclassify SP as an over-the-counter drug. Hence, SP could only be legally sold in pharmacies (Part I drug shops). In many parts of the country, SP was also tolerated in Part II drug stores, though not in general shops. In the study area, the TFDA regulations were enforced, and while the change in malaria policy resulted in a higher treatment efficacy, it also led to an almost 50% decrease in the availability of antimalarials. To improve the availability of antimalarials for home management of malaria, the ACCESS Programme decided to collaborate with a TFDA-supported program that upgrades Part II shops and enables them to sell antimalarials and other essential drugs [33,34].

## Livelihood Assets and the Vulnerability Context

Whether people actually recognize an illness and seek treatment in drug shops or through other health care services depends to a large extent on their access to livelihood assets of the household, the community, and the wider society. These livelihood assets comprise human capital (local knowledge, education, skills), social capital (social networks and affiliations), natural capital (land, water, and livestock), physical capital (infrastructure, equipment, and means of transport) and financial capital (cash and credit) [25]. The availability of these assets is influenced by forces over which people have little control, for instance economy, politics or technology, climatic variability or shocks like floods, draughts, armed conflicts or epidemics. Such factors may be referred to as their vulnerability context.

In the study area of the ACCESS Programme, the Kilombero Valley in southeastern Tanzania, the natural environment increases people's vulnerability to health risks [5]. Malaria is highly endemic, transmission is intense and perennial, and malaria is the predominant cause of morbidity and mortality. Large parts of the valley are flooded during the rain season from November to May. Most of the 517,000 people living in the 109 villages (2002) rely on subsistence agriculture. Labor-intensive rice farming on distant fields in the floodplain forces many families to move to their farming sites during the cultivation period (M. W. Hetzel et al., unpublished data). Already in the village, families face many difficulties in gaining access to the resources necessary for malaria prevention and case management, but even more so in the farming sites [35].

For nearly all members of the study communities, land is the backbone of their livelihood (natural capital) (I. Mayumana, unpublished MA thesis). To raise cash for renting bicycles, buying drugs, or paying treatment expenses (financial capital), farmers have to tap household savings, sell food stock, borrow from local money lenders, and work as causal laborers. Family members and relatives take sick children to health care services, buy drugs, and provide practical and moral support (social capital). Bicycles feature prominently as an asset enabling treatment seeking (physical capital). Popular and biomedical concepts of malaria nowadays overlap (human capital), probably as a consequence of regular and intensive IEC and social marketing campaigns. During its first phase (2003–2007), the ACCESS Programme invested in social marketing to increase knowledge and awareness of malaria and to promote prompt and appropriate treatment seeking from reliable sources [6]. For the second phase starting in 2008, additional initiatives to facilitate access to livelihood assets are planned, such as support to community health funds and provision of microcredits.

## Health Care Utilization and Quality of Care

Depending on access to health care services and to livelihood assets, people develop multiple and changing health care utilization strategies. They may take no action at all or use different service providers simultaneously or in sequence. However, even if they gain access and health care utilization takes its course, the outcome in terms of health status (as evaluated by experts or by patients), patient satisfaction, and equity (defined as equal access to health care by those in equal need [1]) is subject to the technical quality of care. In a broad sense, technical quality of care includes provider compliance and diagnostic accuracy, safety of the product, and patient compliance (or adherence; see Figure 1).

An ACCESS Programme study to determine the effectiveness and promptness of fever treatment based on caregivers' accounts highlights the impact of quality of care (M. Hetzel et al., unpublished paper). A community survey of a random sample of 318 household identified 80 children under five years of age who had a fever (considered as a proxy for malaria) during the 14 days preceding the interview. The results show that 100% of the sick children were treated with a pharmaceutical drug (an antipyretic or antimalarial), 88% were treated with the recommended antimalarial, 76% received the recommended antimalarial on the same day or the day after the fever started, 43% got the recommended antimalarial on the same day or the day after the fever started in the correct dosage, and only 23% were given the recommended antimalarial on the same day or the day after the fever started, in the right dosage, considering also age and the reported symptoms. The multivariate analysis showed that access to and use of a health facility during the course of the fever increased the chance of receiving one of the recommended antimalarials (SP, amodiaquine, or quinine, according to national guidelines) (*p* = 0.004). On the other hand, antimalarials from health facilities were not more accurately dosed than those obtained from shops. To improve quality of care in health facilities, the ACCESS Programme supported the Council Health Management Teams of the two districts in carrying out refresher training in malaria case-management for health facility staff, followed by strengthening of routine supportive supervision and the implementation of a quality management scheme in all health facilities [6].

## Conclusion

Even the most powerful diagnostic tests, drugs, and vaccines have little public health impact if they do not reach the poor. Providing the goods, as well as the services to deliver them, and ensuring that goods and services are of high quality, are major challenges by themselves, especially in a resource-poor setting. But unless additional efforts are made to enable poor people to gain access to these goods and services, as well as to more basic livelihood assets required to initiate treatment seeking, equitable access remains an empty formula of politicians and experts. This is an aspect of the illness–poverty trap that is often overlooked. While it has been increasingly acknowledged that ill-health contributes to poverty because health costs deplete people's meager resources, it is hardly recognized that people often cannot even gain access to health services because they cannot mobilize critical livelihood resources. This article presents an innovative framework that pulls together the strength of social sciences, public health research, and development studies. Through this combination of perspectives and expertise, a more comprehensive, but structured analysis of access to health care in resource-poor settings can be achieved, which will lead to the identification of key entry points and targeted action for health and poverty alleviation in horizontal community-based approaches.

## Abbreviations

IEC: information, education, and communication
PIOP: policies, institutions, organizations, and processes
SP: sulphadoxine-pyrimethamine
TFDA: Tanzania Food and Drugs Authority
